# A randomized phase 3 study on the optimization of the combination of bevacizumab with FOLFOX/OXXEL in the treatment of patients with metastatic colorectal cancer-OBELICS (Optimization of BEvacizumab scheduLIng within Chemotherapy Scheme)

**DOI:** 10.1186/s12885-016-2102-y

**Published:** 2016-02-08

**Authors:** Antonio Avallone, Maria Carmela Piccirillo, Luigi Aloj, Guglielmo Nasti, Paolo Delrio, Francesco Izzo, Elena Di Gennaro, Fabiana Tatangelo, Vincenza Granata, Ernesta Cavalcanti, Piera Maiolino, Francesco Bianco, Pasquale Aprea, Mario De Bellis, Biagio Pecori, Gerardo Rosati, Chiara Carlomagno, Alessandro Bertolini, Ciro Gallo, Carmela Romano, Alessandra Leone, Corradina Caracò, Elisabetta de Lutio di Castelguidone, Gennaro Daniele, Orlando Catalano, Gerardo Botti, Antonella Petrillo, Giovanni M. Romano, Vincenzo R. Iaffaioli, Secondo Lastoria, Francesco Perrone, Alfredo Budillon

**Affiliations:** Multidisciplinary Treatment Unit, Gastrointestinal Medical Oncology Unit, Istituto Nazionale per lo Studio e la Cura dei Tumori “Fondazione Giovanni Pascale” – IRCCS, Napoli, Italy; Clinical Trials Unit, Istituto Nazionale Tumori “Fondazione G. Pascale” – IRCCS, Napoli, Italy; Nuclear Medicine Unit, Istituto Nazionale per lo Studio e la Cura dei Tumori “Fondazione Giovanni Pascale” – IRCCS, Napoli, Italy; Gastrointestinal Medical Oncology Unit, Istituto Nazionale per lo Studio e la Cura dei Tumori “Fondazione Giovanni Pascale” – IRCCS, Napoli, Italy; Colorectal Surgery Unit, Istituto Nazionale per lo Studio e la Cura dei Tumori “Fondazione Giovanni Pascale” – IRCCS, Napoli, Italy; Hepatobiliary Surgery Unit, Istituto Nazionale per lo Studio e la Cura dei Tumori “Fondazione Giovanni Pascale” – IRCCS, Napoli, Italy; Experimental Pharmacology Unit, Istituto Nazionale per lo Studio e la Cura dei Tumori “Fondazione Giovanni Pascale” – IRCCS, Napoli, Italy; Pathology Unit, Istituto Nazionale per lo Studio e la Cura dei Tumori “Fondazione Giovanni Pascale” – IRCCS, Napoli, Italy; Radiology Unit, Istituto Nazionale per lo Studio e la Cura dei Tumori “Fondazione Giovanni Pascale” – IRCCS, Napoli, Italy; Laboratory Medicine Unit, Istituto Nazionale per lo Studio e la Cura dei Tumori “Fondazione Giovanni Pascale” – IRCCS, Napoli, Italy; Pharmacy Unit, Istituto Nazionale per lo Studio e la Cura dei Tumori “Fondazione Giovanni Pascale” – IRCCS, Napoli, Italy; Gastrointestinal Surgery Unit, Istituto Nazionale per lo Studio e la Cura dei Tumori “Fondazione Giovanni Pascale” – IRCCS, Napoli, Italy; Vascular Access Unit, Istituto Nazionale per lo Studio e la Cura dei Tumori “Fondazione Giovanni Pascale” – IRCCS, Napoli, Italy; Endoscopy Unit, Istituto Nazionale per lo Studio e la Cura dei Tumori “Fondazione Giovanni Pascale” – IRCCS, Napoli, Italy; Radiotherapy Unit, Istituto Nazionale per lo Studio e la Cura dei Tumori “Fondazione Giovanni Pascale” – IRCCS, Napoli, Italy; Medical Oncology Unit, San Carlo Hospital, Potenza, Italy; Department of Clinical Medicine and Surgery, University Federico II, Naples, Italy; A.O. Valtellina e Valchiavenna, Sondrio, Italy; Medical Statistics Unit, Second University, Naples, Italy; Istituto Nazionale per lo Studio e la Cura dei Tumori “Fondazione Giovanni Pascale” – IRCCS, Napoli, Italy

**Keywords:** Colorectal cancer, Bevacizumab, Oxaliplatin, Vessel normalization, FDG-PET, Biomarkers for anti-angiogenic therapy

## Abstract

**Background:**

Despite the improvements in diagnosis and treatment, colorectal cancer (CRC) is the second cause of cancer deaths in both sexes. Therefore, research in this field remains of great interest. The approval of bevacizumab, a humanized anti-vascular endothelial growth factor (VEGF) monoclonal antibody, in combination with a fluoropyrimidine-based chemotherapy in the treatment of metastatic CRC has changed the oncology practice in this disease. However, the efficacy of bevacizumab-based treatment, has thus far been rather modest. Efforts are ongoing to understand the better way to combine bevacizumab and chemotherapy, and to identify valid predictive biomarkers of benefit to avoid unnecessary and costly therapy to nonresponder patients. The BRANCH study in high-risk locally advanced rectal cancer patients showed that varying bevacizumab schedule may impact on the feasibility and efficacy of chemo-radiotherapy.

**Methods/Design:**

OBELICS is a multicentre, open-label, randomised phase 3 trial comparing in mCRC patients two treatment arms (1:1): standard concomitant administration of bevacizumab with chemotherapy (mFOLFOX/OXXEL regimen) vs experimental sequential bevacizumab given 4 days before chemotherapy, as first or second treatment line. Primary end point is the objective response rate (ORR) measured according to RECIST criteria. A sample size of 230 patients was calculated allowing reliable assessment in all plausible first-second line case-mix conditions, with a 80 % statistical power and 2-sided alpha error of 0.05. Secondary endpoints are progression free-survival (PFS), overall survival (OS), toxicity and quality of life. The evaluation of the potential predictive role of several circulating biomarkers (circulating endothelial cells and progenitors, VEGF and VEGF-R SNPs, cytokines, microRNAs, free circulating DNA) as well as the value of the early [^18^F]-Fluorodeoxyglucose positron emission tomography (FDG-PET) response, are the objectives of the traslational project.

**Discussion:**

Overall this study could optimize bevacizumab scheduling in combination with chemotherapy in mCRC patients. Moreover, correlative studies could improve the knowledge of the mechanisms by which bevacizumab enhance chemotherapy effect and could identify early predictors of response.

**EudraCT Number:** 2011-004997-27

**Trial registration:**

ClinicalTrials.gove number, NCT01718873

## Background

### Bevacizumab in the treatment of colorectal cancer

Colorectal cancer (CRC) is the second most common cancer and the third cause of cancer deaths in both sexes, comprising approximately 13 % of all new cancer diagnoses in Europe [1]. Approximately, 15 % of patients with CRC are diagnosed with metastatic disease, and a further 40–50 % will develop metastases during the course of their disease. Despite the improvements in diagnosis and treatment, unresectable metastatic colorectal cancer (mCRC) remains an incurable disease with a 5-year survival rate of approximately 10 % [1]. Therefore, research in this field remains of great interest. In the last ten years angiogenesis has emerged as a crucial hallmark of cancer development, becoming a key target for cancer treatment [2, 3]. Tumor angiogenesis is characterized by structural and functional abnormalities of vasculature with a relatively inefficient blood supply and the vascular endothelial growth factor (VEGF) has a crucial role in these abnormalities [4, 5]. Bevacizumab, a humanized monoclonal antibody that inhibits tumor angiogenesis by blocking VEGF, has been the first antiangiogenic agent approved for the treatment of cancer. Its approval in combination with a fluoropyrimidine-based chemotherapy has changed oncology practice of mCRC. However, despite promising preclinical results, the objective response rates (ORR) and survival benefits of bevacizumab have thus far been rather limited, stimulating interest in developing more effective ways to combine the drug and the chemotherapy [6]. Moreover, the magnitude of the benefit is heterogeneous across trials and seems to be affected by the chemotherapeutic regimen with which bevacizumab is partnered. In the NO16966 trial, bevacizumab in combination with XELOX or FOLFOX-4 in the first line treatment of patients with mCRC, did not increase the ORR (38 % vs 38 %), did not significantly prolong the overall survival (OS) and, although the progression-free survival (PFS) was significantly improved (HR = 0,83; *P* = 0.0023), the improvement was clinically modest (only 1,4 months) and markedly less than that found in the AVF2107 trial, where the drug was combined with IFL [7, 8]. The modest benefit in survival observed in the NO16966 trial might be explained by the fact that the majority of patients did not continue the treatment until progression, however the lack of impact of bevacizumab on RR would remain unclear [7].

Other two randomized phase 3 trials confirmed that bevacizumab combined with first-line chemotherapy is not superior to chemotherapy alone [9, 10].

Overall, these data suggest that additional investigation are needed to improve bevacizumab antitumor effect and to identify valid predictive biomarkers for patient selection [11].

### Mechanism of action of bevacizumab

Despite extensive preclinical and clinical studies little is known about the mechanism (or mechanisms) of action of bevacizumab, especially when co-administered with chemotherapy. Emerging data suggest that bevacizumab, not only blocks the growth of new blood vessels [12], but also induces pharmacodynamic changes that support the “vessel normalization” hypothesis [4, 13]. As the abnormal tumor vasculature produces elevated interstitial fluid pressure, tumor hypoxia and reduces blood flow and perfusion, this prevents the delivery of anticancer drugs and may cause resistance to chemotherapy [4]. The treatment with bevacizumab can normalize the tumor vasculature, resulting in more efficient drug and oxygen delivery to cancer cells [4, 14]. However, this process of vascular normalization seems to be transient and with a relatively narrow therapeutic window. Chemotherapy and/or radiation therapy given during this transient “window of normalization” may be more effective [4, 15–17]. Preclinical eivdences suggest that bevacizumab needs at least 4–5 days to reduce tumor interstitial fluid pressure and increase tumor oxygenation [15–17]. In this scenario, the optimal scheduling of the anti-angiogenic agent with the cytotoxic therapies would become the major determinant of the overall therapeutic effect.

## Rationale

### Critical role of bevacizumab scheduling in combination with pre-surgical chemo-radiotherapy in locally advanced rectal cancer: the BRANCH study

Based on the “vessels normalization” hypothesis, we performed a non-randomized, non-comparative phase 2 study to assess the safety and the activity of a traditional concurrent and an experimental sequential (4 days before chemo-radiotherapy) administration of bevacizumab, with preoperative chemo-radiotherapy, in magnetic resonance imaging (MRI)-defined high-risk locally advanced rectal cancer (LARC) patients (BRANCH study) [18]. Patients received three biweekly cycles of pre-operative oxaliplatin (OXA), raltitrexed (RTX), fluorouracil (5FU), and folinic acid (LFA), during pelvic radiotherapy (RT). Bevacizumab was given 2 weeks before the start of chemo-RT and on the same day of chemotherapy for three cycles (concomitant-schedule) or 4 days prior to the first and second cycle of chemotherapy (sequential-schedule). The primary end point was pathological complete tumor regression (TRG1) rate. To establish the sample size, the Simon’s two-stage design was applied. Setting α and β errors at 0.05 and 0.20, respectively, and defining as the minimum activity of interest (p0) a TRG1 rate of 30 %, in order to accept the alternative hypothesis (p1) of a TRG1 rate ≥ 50 %, at least 6 TRG1 in the first 15 patients and at least 19 TRG1 among a total of 46 patients would need to be reported in the first and second stage, respectively. The accrual in the concomitant-schedule group was early stopped due to lack of activity (two TRG1 out of 16 patients). Conversely, a TRG1 rate of 50 % (95 % CI 35–65 %) was obtained with the sequential-schedule among the 46 enrolled patients. In this group, the 5-year probability of progression free-survival (PFS) and OS were 80 % (95 % CI 66–89 %) and 85 % (95 % CI 69–93 %), respectively. Neutropenia was the most common grade ≥3 toxicity with both schedules, but it was less frequent with the sequential than the concomitant-schedule (30 % vs. 44 %). Such result have been recently confirmed by data from preclinical studies, showing that the sequential delivery of an anti-angiogenic therapy followed by chemotherapy induces less bone marrow toxicity than concomitant administration [19].

These results highlight the relevance of bevacizumab scheduling to optimize its combination with chemo-RT.

### Circulating biomarkers and imaging study

The identitification of validated predictive biomarkers of anti-angiogenic therapy efficacy remains an unmeet need. Recent studies suggested that bevacizumab could induce a chemosensitization also inhibiting the rapid tumor cell repopulation that can take place between successive chemotherapy administrations [20]. Such process involves several cytokines and the mobilitation of the circulating endothelial cells (CEC) and endothelial progenitor cells (EPC), which are rare cell subsets, detectable in peripheral blood, cord blood and bone marrow that act as key players in the maintenance of the endothelial homeostasis. CEC, characterized by mature endothelial features, detach from vessel walls, following vascular damage or its physiological turnover, and become circulating cells [21]. On the other hand, EPC, characterized by an immature phenotype, are bone marrow-resident cells, mobilized upon specific stimulation, including chemotherapy, that, once in the bloodstream, are involved in the endothelial repair or remodeling [20, 22]. Over the past decade, several reports demonstrated a high CECs count in the peripheral blood of cancer patients at diagnosis; therefore, it might be a promising tool for patients who would benefit from anti-angiogenic therapies [21, 23, 24]. On the other hand, chemotherapy can mobilized EPCs from bone marrow that may contribute to neovascularization; therefore, an early anti-angiogenic therapy in combination with chemotherapy could enhance treatment effect [20, 25]. Functional analyses of early clinical study in LARC patients have confirmed that bevacizumab normalizes tumor vasculature and decreases CECs and EPCs count [26, 27]. A recent study confirmed a host response with EPC mobilization in colorectal cancer patients during FOLFOX adjuvant chemotherapy, which resulted significantly inhibited by the addition of bevacizumab to FOLFOX [28]. Interestingly, in the BRANCH study we showed that baseline CEC counts were higher in responding (TRG1-2) vs non-responding (TRG3-4) patients and that CEC counts significantly reduced during treatment only in the responders [29].

Although different flow cytometry methods for CEC and EPC characterization have been published so far, no one has reached consistent conclusions. Therefore consensus guidelines with respect to CEC and EPC identification and quantification need to be established. On this regard, we have been recently involved in a multicentre study carrying out a deep investigation of CEC and EPC phenotypes and optimizing a simple and reliable polychromatic flow cytometry single panel method that allows the assessment of these cellular populations in the perypheral blood of healthy donors. Interestingly, our data also suggest that the antigen profile for the identification of endothelial progenitors circulating in the bloodstream might be redefined [30].

Some recent reports have evidenced that several circulating cytokines might be modulated in cancer patients undergoing anti-VEGF therapy, correlating with antitumor efficacy [31, 32]. On this regard, multiplex technologies offer a noninvasive, easy and convenient method of simultaneously assessing a large number of biologically relevant citochine and angiogenic factor from small plasma volumes.

It has been recently demonstrated that single nucleotide polymorphisms (SNP) in VEGF, VEGF-R and other genes involved in angiogenesis, alter their proteins concentrations influencing the process of angiogenesis. Moreover, VEGF SNPs seems to play an important role in the risk of recurrence, prognosis and survival of colorectal cancer as well as in the response to bevacizumab treatment [11, 33–35].

The high stability of circulating free DNA (cfDNA) and miRNA in the plasma of patients with cancer, suggest the possibility to identify innovative predictive biomarkers of benefit or resistance for anti-angiogenic therapy. Blood-based molecular tests, such as the so-called “liquid biopsy”, can be used to detect cancer-specific DNA alterations in plasma of colorectal cancer patients with very high sensitivity and might be applied for monitoring treatment response and assessing minimal residual disease [36]. Moreover, recent studies have shown that the expression of microRNAs (miRNAs) in the plasma of patients with colorectal cancer could be used for diagnosis and prognosis [37]. MiRNAs may also mediate the regulation of “switch” angiogenesis [38]. There are also experimental evidence demonstrating a possible use of miRNA expression for the prediction of response to various chemotherapy [39].

Our group has previously reported that early metabolic change evaluated by [^18^F]-Fluorodeoxyglucose positron emission tomography (FDG-PET) is able to predict pathologic tumor response and outcome in rectal cancer [40, 41] and in mCRC [42]. Interestingly, in the BRANCH study we have also observed a greater early reduction (11 days after chemo-RT) of median tumor metabolic volume, evaluated by FDG-PET in the sequential-schedule than in the concomitant-schedule [43].

## Methods/Design

OBELICS is a prospective, multicentre, open-label, randomised, phase 3 trial evaluating the optimization of bevacizumab scheduling in combination with chemotherapy in mCRC patients. The study includes an explorative analysis of the potential prognostic or predictive role of several circulating biomarkers as well as of early FDG-PET evaluation. With the aim of improving the knowledge of the mechanisms by which bevacizumab enhances chemotherapy effect and of identifying early predictors of treatment response/resistence.

### Objectives

The primary objective of the study is to assess whether an experimental schedule of bevacizumab, given sequentially instead of concomitantly with oxaliplatin regimen (mFOLFOX/mOXXEL), can improve treatment activity (in terms of objective response rate) in patients with mCRC.

Secondary objectives are to evaluate the impact of the experimental schedule on PFS, OS, toxicity and quality of life.

Moreover, the study has the objective of validating the prognostic and predictive role of the early metabolic response evaluated by FDG-PET (11 days after the start of the first cycle of chemotherapy in both arms, that is 15 days after the first administration of bevacizumab in the experimental arm).

Exploratory secondary objective is to evaluate the prognostic and predictive value of: a) CEC and EPC counts on patient blood samples at baseline and at different time points during and after treatment; b) cytokine and circulating angiogenic factors plasma levels on patient blood samples at baseline and at different time points during and after treatment; c) miRNAs on patient blood samples at baseline and at different time points during and after treatment; d) single nucleotide polymorphisms (SNPs) of VEGF and VEGF receptor (VEGF-R) on patient blood samples; e) white blood cells counts at 24 h after the first administration of bevacizumab on patient blood samples; f) genetic alterations on tumor tissues and/or on cfDNA.

### Ethical aspects

The procedures set out in this study protocol are designed to ensure that the principles of the Good Clinical Practice guidelines of the International Conference on Harmonization (ICH) and the Declaration of Helsinki are respected in the conduct, evaluation and documentation of this study. The study was approved by the Ethical Committee of the National Cancer Institute of Naples, Italy. Patients provide written informed consent for participating in the study and for allowing to collect tissue and blood samples.

### Study design

**Fig. 1:**
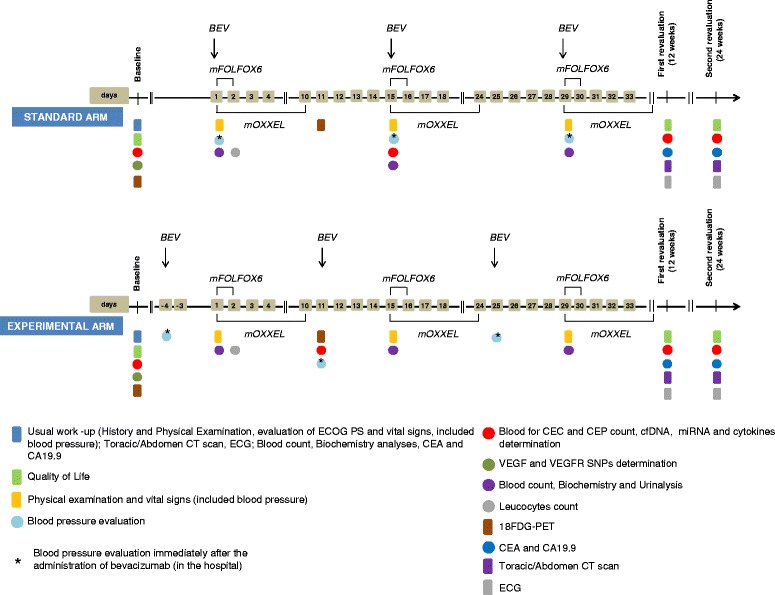
Schematic timeline of study procedures

OBELICS is a two-arm phase 3 trial comparing in mCRC patients (1:1): concurrent administration of bevacizumab in combination with modified FOLFOX-6 regimen (mFOLFOX-6) or modified OXXEL regimen (mOXXEL), in which bevacizumab is administered the same day as oxaliplatin, (standard arm); and sequential administration of bevacizumab with the same chemotherapeutic regimens, in which bevacizumab is administered 4 days before oxaliplatin at each cycle (experimental arm) (Fig. 1). Oxaliplatin regimen (mFOLFOX/mOXXEL) is chosen according to local clinical practice at the beginning of the study.

In both arms, the patients not progressing after 12 cycles (24 weeks) of treatment, stop chemotherapy and continue maintenance bevacizumab (7.5 mg/kg every 3 weeks) until progression, unacceptable toxicity or patient’s choice to stop.

Primary end point is objective ORR according to RECIST criteria version 1.1.

Study sample size is defined according to the ORR expected in the control arm; it varies as a function of the proportion of patients in first and in second line of treatment enrolled in the study. Assuming 40 % as the expected first line ORR and 20 % as the expected second line one, the sample size is calculated within three different expected ORR: 35 % (75 % as first line and 25 % as second line), 30 % (50 and 50 % respectively) and 25 % (25 and 75 % respectively). In the Table 1, the expected sample size is reported, under the three hypothesized conditions, fixing an odds ratio of 2.25, 80 % statistical power and 2-sided alpha error of 0.05. We have also reported the auspicated ORRs, in the experimental arm, and the corresponding relative risks, ranging from 1.6 to 1.7. As expected, odds ratio represents an overestimation of relative risk, and the extent of overestimation increases with increasing the expected ORR. Therefore, a sample size of 220–230 patients will allow reliable assessment in all plausible case-mix conditions.

**Table 1:** Plausible case mix conditions

% expected response in standard arm	# patients	% expected response in experimental arm	Relative risk (experimental *vs* standard)
35	201	54,8 %	1,57
30	209	49,1 %	1,63
25	230	42,9 %	1,71

### Patient selction criteria

#### Inclusion criteria

Patients are eligible if ≥18 and ≤ 75 years old, diagnosed with metastatic adenocarcinoma of colon or rectum (Stage IV), regardless of RAS mutational status, have at least one measurable target lesion (according to the RECIST criteria), have an ECOG Performance Status ≤1 at study entry and a life expectancy > 3 months. Moreover, they have to had adequately recovered from recent surgery (at least 28 days after a major surgery or biopsy) and consenting to use effective contraception if the risk of conception exist. All the patients sign a written informed consent.

#### Exclusion criteria

Patients are excluded if they have received more than one line of treatment for metastatic disease, or a previous treatment with bevacizumab or oxaliplatin (a previous treatment with fluoropirymidines, folic acid, irinotecan or cetuximab is allowed); have a primary cancer that produces stenosis or full-thickness wall infiltration not resolved by stent placement or surgery; use regularly NSAID or aspirin (more than 325 mg/die) or anticoagulants at therapeutic dose; have bleeding diathesis or pre-existing coagulopathy; have known or suspected brain metastases (determined exclusively in the presence of at least one clinical symptom); have inadequate bone marrow, liver or renal function (Neutrophils < 2000/mm^3^ or platelets < 100.000/mm^3^ or haemoglobin <9 gr/dl; creatinine levels of >1.5 time the upper normal limit UNL; GOT and/or GPT > 2.5 time the UNL and/or bilirubin >1.5 time the UNL in absence of liver metastasis; GOT and/or GPT > 5 time the UNL and/or bilirubin > 3 time the UNL in presence of liver metastasis; have had any other malignancy other than non-melanomatous skin cancer, or carcinoma in situ of the cervix In the last 5 years; have congestive heart failure, recent ischemic coronary disease (last 12 months), uncontrolled arrhythmia, uncontrolled hypertension, active or uncontrolled infection, or any other serious uncontrolled medical disorder that in the opinion of the investigator would impair the ability of the subject to receive protocol therapy. Pregnant or lactating women are excluded. Moreover, patients with a history or current evidence on physical examination of central nervous system disease or peripheral neuropathy > grade 1 (CTCAE v. 4.0), or are unable to comply with follow-up are excluded.

### Treatment plan

In the standard arm bevacizumab (5 mg/kg) is administered as 20- to-30 min intravenous infusion before oxaliplatin on day 1 of each cycle of mFOLFOX-6 regimen (oxaliplatin 85 mg/m^2^ i.v. infusion on day 1 followed by levo-folinic acid 200 mg/m^2^ i.v. infusion followed by i.v. bolus 5-fluorouracil 400 mg/m^2^, and a 46-hour i.v. infusion of 5-fluorouracil 2400 mg/m^2^) or mOXXEL regimen (oxaliplatin 85 mg/m2 i.v. infusion on day 1 plus oral capecitabine 1000 mg/m^2^ twice daily on days 1 to 10) every 2 weeks for 12 cycles (24 weeks). In the experimental arm bevacizumab (5 mg/kg) is administered 4 days (day −4) before oxaliplatin on day 1 of each cycle of mFOLFOX-6 or mOXXEL regimen (at same doses used in the standard arm) every 2 weeks for 12 cycles (24 weeks) (Fig. 1).

Thereafter, in both arms, patients who are progression free after 12 cycles (24 weeks) of treatment continue maintenance bevacizumab (7.5 mg/kg every 3 weeks) ± fluoropyrimidines until disease progression or unacceptable toxicity.

Surgery may be carried out in case of appropriate tumour reduction is evident at response evaluation. Resectability has to be evaluated by a multidisciplinary review team and the decision regarding post-surgery chemotherapy is at the discretion of the investigators, according to their local clinical practice.

In cases of prespecified adverse events, treatment modifications are permitted as follow: a 25 % fluoropyrimidines dose reduction is applied in subsequent cycles in case of grade ≥ 3 of haematologic or non-haematologic toxicities (except for alopecia); at the second appearance of these side effects a dose reduction of 50 % of fluoropyrimidines may be applied; after a further grade ≥ 3 toxicity or after the first appearance of grade ≥ 3 sensory neuropathy, a 25 % oxaliplatin dose reduction is planned; at the second appearence of grade ≥ 3 sensory neuropathy a 50 % oxaliplatin dose reduction is also planned; otherwise chemotherapy is permanently discontinued. No profilactic use of G-CSF or eritropoyetin is planned. No dose-reduction is planned for bevacizumab.

Bevacizumab has to be permanently discontinued in patients who develop any one of the following toxicities: gastrointestinal perforation, grade ≥ 3 thromboembolism, grade 4 hemorrhage, grade ≥ 3 hypertension or proteinuria, congestive heart failure.

An anti-hypertensive treatment (ACE inhibitors and/or calcium antagonists at standard doses) should be undertaken in case of grade ≥ 2 hypertension (recurrent or persistent), or in case of an increase symptomatic > 20 mmHg (diastolic) or in case of a pressure > 150/100 mm Hg if normal at baseline.

### Assessment and procedures

Assessment and procedures, including those for exploratory objectives (see below) are illustrated in Fig. 1.

#### Toxicity evaluation criteria

Toxicity is graded according to the Common Terminology Criteria for Adverse Events (CTCAE) of the National Cancer Institute, version 4.0, June 14, 2010.

Adverse events are assessed at the following times: baseline (within 3 weeks before the treatment start), weekly (blood count) and beweekly (biochemistry, physical examination, ECOG performance status and vital signs including blood pressure) during the treatment. ECG is performed at weeks 12 and 24 weeks from randomization.

#### Response evaluation criteria

Response is assessed at week 12 and 24 from randomization, and every 3 months thereafter, until disease progression, by repeating: CT scan of chest, abdomen and pelvis; CEA, CA 19.9; any other tests having resulted positive during baseline staging. Objective response will be categorized according to Response Evaluation Criteria in Solid Tumors (RECIST) v. 1.1. An independent blinded central review of radiologic examinations will be performed.

### FDG-Positron Emission Tomography (PET) imaging

FDG PET-CT scans are planned at baseline (within 3 weeks before treatment start) and 11 days after the start of the first cycle of chemotherapy in both arms (that is 15 days after the first administration of bevacizumab in the experimental arm).

Patients have to be fasted for at least 6 h and blood glucose level have to be less than 150 mg/dL. FDG-PET images are reconstructed using iterative reconstruction and normalized for injected dose and patient body weight. Image analysis is performed utilizing a semi-automatic region-of-interest (ROI) drawing software package where a three-dimensional region is drawn around the area of increased uptake. Threshold values are adjusted in order to encompass the area of increased uptake visually.

For each tumor volume, the following parametres are calculated: a) SUV = (measured activity concentration [Bq/mL])/(injected activity [Bq]/body weight [kg] 1000); b) SUV-max = the maximum pixel value measured in the visualized lesion; c) SUV-mean = the average activity values in the ROIs; d) TLG (Total Lesion Glycolysis) = SUV-mean x metabolic tumor volume (mm^3^).

On the basis of these parameters, the following indicator are calculated for each patients, in order to assess metabolic response: 1) SUVmax = the highest SUVmax value among all the evaluable lesions; 2) SUVmaxsum = the sum of the SUVmax value of all the evaluable lesions; 3) TLGmax = the highest TLG value among all the evaluable lesions; 4) TLGsum = the sum of the TLG value of all the evaluable lesions.

Metabolic response is calculated by measuring changes reported at the post treatment examination (at day11 after the start of the first cycle of chemotherapy in both arms) compared to the baseline test. For all the indicators the change is calculated as: Δ = (value-post – value-baseline)/value-baseline × 100.

Consistent with previous studies [40–42] the threshold to define a patient as responder according to change of SUV or TLG indicators is ≥ 50 %. Therefore, patients with any change above these thresholds are defined as non-responder. This value will be validated within this study for its predictive ability of survival outcome. Further thresholds will be eventually explored only in case of failure (lack of predictive ability) of the proposed validation.

### Biomarkers

Peripheral blood samples are collected at baseline, on day 15th after the start of the first administration of bevacizumab in both arms (that is 11 days after the first chemotherapy administration in the experimental arm) at weeks 12 and 24, after randomization and at the progression of the disease (PD). Further blood samples are collected before surgery in patients undergoing resection of metastases. CEC and EPCs counts will be analyzed as surrogate marker of tumor angiogenesis at baseline, on day 15th after the start of the first administration of bevacizumab in both arms at weeks 12 and 24 and at PD, by flow cytometry [30].

Analysis of polymorphisms in VEGF and VEGFR genes will be evaluated on DNA from peripheral blood collected at baseline and aliquoted and stored at −80 °C. After DNA extraction the expression of the indicated polymorphisms will be evaluated by RT-PCR using specific TAqman probes.

Analysis of circulating angiogenic factors and cytokines will be performed on peripheral blood samples aliquoted and stored at −80 °C (collected at each of the above reported time-points for CEC and EPCs evaluation) by Bio-Plex™ thecnology.

Analysis of miRNA will be performed on peripheral blood samples aliquoted and stored at −80 °C (collected at each of the above reported time-points for CEC and EPCs evaluation) by droplet digital PCR (ddPCR) technology [44].

Analysis of cfDNA mutations will be performed on peripheral blood samples aliquoted and stored at −80 °C (collected at each of the above reported time-points for CEC and EPCs evaluation and before metastasis surgery). After genome equivalent absolute quantification mutation analysisis will be performed by BEAMing, Droplet digital PCR analysis or Next Generation Sequencing analysis [45].

Finally we have planned to collect for a centralised revision at the National Cancer Institute two tumor samples for all patients enrolled (primary tumor and eventual resected metastases), to assess RAS and BRAF mutations. We will also evaluate on collected tumor samples other potential predictive markers of response.

### Quality of life assessment

Quality of Life is assessed by the EORTC QLQ-C30, v. 3.0 questionnaire that are completed by patients at baseline and at week 12 and 24 during treatment, in both arms [46].

### Statistical analysis

All analyses will be performed according to an intention to treat strategy.

ORR is defined as the number of complete plus partial response divided by the total of patients enrolled in each comparison arm. ORR will be described by 2x2 contingency tables and statistical significance of the possible difference will be estimated by chi-square test. The difference between RR in the two arms will be estimated with 95 % confidence interval.

Progression Free Survival (PFS) is defined as the time from randomization to the date of progression, the date of death without progression or the date of the last follow-up information available, whichever occurred first. Curves will be drawn with the Kaplan-Meier product-limit method. Statistical significance will be calculated by a model of multivariable analysis considering stratification factors as covariates.

Overall Survival (OS) is defined as the time from randomization to the date of death or the date of termination of the trial (for patients alive at the time end of the study), or the date of the last follow-up information available (for patients loss before the trial end date). Curves will be drawn with the Kaplan-Meier product-limit method. Statistical significance will be calculated by a model of multivariable analysis considering stratification factors as covariates.

For each patient and type of toxicity, the worst degree suffered during the treatment will be described. Patients who will have not received assigned treatment will be excluded. Statistical analysis will be performed by contingency tables and statistical significance of the possible differences between the treatment groups will be calculated with a linear permutation test accounting for ordinal nature of data (linear rank test).

Biomarkers data will be conducted with the aim of hypothesis generation. First of all, a complete description of data from biological and pharmacogenomic studies will be done. For biomarkers that might change over time as a consequence of treatment, levels before and after treatment will be compared with appropriate statistical tests, based on the type of data. *P* values ≤0.05 will be considered significant, and no adjustment is planned for multiple comparisons due to the exploratory nature of the analysis.

### Registration and data collection procedures

Procedures for registration, randomization and data collection are centralized and web-based through the on-line platform of the Clinical Trials Unit of the NCI of Naples (http://www.usc-intnapoli.net.) Biological analyses are centralized at the Experimental Pharmacology Unit of the NCI of Naples. Randomization is performed with a minimization procedure that accounts for the following parameters as strata: center, ECOG performance status (0 vs 1), previous chemotherapy for advanced disease (yes vs no) and number of metastatic sites (1 vs more).

## Discussion

The goal of OBELICS study is to evaluate the optimization of bevacizumab scheduling in combination with chemotherapy in mCRC patients by comparing in a multicentre randomised phase 3 trial the traditional concomitant administration of bevacizumab in combination with chemotherapy (mFOLFOX/OXXEL regimen), with an experimental schedule, defined on the basis of “normalization hypothesis”, in which bevacizumab is given 4 days before chemotherapy.

Since there is an unmet need for pharmacodynamic and predictive biomarkers of benefit for anti-angiogenic drugs we will explore the potential predictive role of several circulating biomarkers as well as of the early metabolic response to improve the knowledge of the mechanisms by which bevacizumab enhance chemotherapy effect and to identify early predictors of response. In particular, we plan to analyze, at multiple time points, a complete kinetic profile of several potential biomarkers on peripheral blood samples, considering that cancer have a dynamic nature and that the possibility of evaluating tumor changes by repeated biopsies is limited by patients discomfort and tumor heterogeneity.

The SNPs of the VEGF gene, the count (baseline and during treatment) of CECs and their progenitors EPCs, together with a broad profile of cytokines and angiogenic factors, could help to select the patients who are most likely to benefit from these high-cost therapies and/or to identify possible mechanisms of resistance. Multiplex technologies offer a noninvasive, easy and convenient method of simultaneously assessing a large number of biologically relevant citochine and angiogenic factor from small plasma volumes. Moreover, the high stability of cfDNA and miRNA in the plasma of patients with cancer and their correlation with the expression in the tumor, suggest the possibility to identify innovative predictive biomarkers of benefit for anti-angiogenic therapy.

Pursuing imaging (FDG-PET) and circulating biomarkers early after treatment initiation might be a fruitful approach, as many of the biomarker changes occur rapidily after the onset of therapy and the ability to identify these changes early may allow to tailor the therapy and to discontinue early ineffective treatment.

Overall, the outcome of this correlative studies could help to optimize anti-angiogenic therapy in CRC patients.

### Trial sponsorship

The study is a multicentre non-profit, independent investigator initiated trial supported by a grant of the Ministry of Health (RF-2009-1539464). Istituto Nazionale Tumori Fondazione G. Pascale, Napoli, Italy, will take out insurance coverage for trial participants.

## Abbreviations

5FU: fluorouracil
CEC: circulating endothelial cells
cfDNA: circulating free DNA
CRC: colorectal cancer
CT: computed tomography
CTCAE: common terminology criteria for adverse events
ddPCR: droplet digital PCR
EPC: endothelial progenitor cells
FDG-PET: [^18^F]-Fluorodeoxyglucose positron emission tomography
ICH: International Conference on Harmonization
LARC: locally advanced rectal cancer
LFA: folinic acid
mCRC: metastatic colorectal cancer
mFOLFOX-6: modified FOLFOX-6 regimen
miRNAs: microRNAs
mOXXEL: modified OXXEL regimen
MRI: magnetic resonance imaging
MTD: maximum tolerated dose
ORR: overall response rate
OS: overall survival
OXA: oxaliplatin
PD: progression disease
PFS: progression free-survival
RECIST: response evaluation criteria in solid tumors
ROI: region-of-interest
RT: radiotherapy
RTX: raltitrexed
SNP: single nucleotide polymorphisms
SUV: standardized uptake value
TLG: total lesion glycolysis
VEGF: vascular endothelial growth factor
VEGF-R: vascular endothelial growth factor receptor
WBC: white blood cell
